# Longitudinal changes of oxidative stress and PON1 lactonase activity and status in older pregnant women undergoing assisted reproductive technology: a prospective nested case-control study

**DOI:** 10.1186/s12958-023-01139-w

**Published:** 2023-10-26

**Authors:** Chenyu Jiang, Meng Chen, Yujie Wu, Huai Bai, Xinghui Liu, Ping Fan

**Affiliations:** 1 Department of Obstetrics and Gynecology, West China Second University Hospital, Sichuan University, Chengdu, Sichuan 610041 China; 2 Laboratory of Genetic Disease and Perinatal Medicine, Key Laboratory of Birth Defects and Related Diseases of Women and Children, Ministry of Education, West China Second University Hospital, Sichuan University, Chengdu, Sichuan 610041 China

**Keywords:** Advanced maternal age, Assisted reproductive technology, Paraoxonase 1, Lactonase activity, Genetic polymorphism, Oxidative stress, Homocysteine

## Abstract

**Background:**

Childbearing in women with advanced maternal age (AMA) has increased the need for artificial reproductive technology (ART). ART and oxidative stress are associated with many pregnancy complications. Paraoxonase (PON) 1 is one of the key components responsible for antioxidative activity in high-density lipoprotein (HDL). This study aimed to investigate the longitudinal changes of oxidative stress and PON1 lactonase activity and status in older women undergoing ART.

**Methods:**

This prospective nested case-control study included 129 control and 64 ART women. Blood samples were obtained respectively at different stages of pregnancy. PON1 level and lactonase activity were assessed using 7-O-diethylphosphoryl-3-cyano-4-methyl-7-hydroxycoumarin (DEPCyMC) and 5-thiobutyl butyrolactone (TBBL) as a substrate, respectively. A normalized lactonase activity (NLA) was estimated based on the ratio of TBBLase to DEPCyMCase activity. Serum total oxidant status (TOS), total antioxidant capacity (TAC), malondialdehyde (MDA), homocysteine (HCY), *PON1* C-108T and Q192R genetic polymorphisms, and metabolic parameters were analyzed.

**Results:**

Lactonase activity and level of PON1 gradually decreased with pregnancy progression, while glycolipid metabolism parameters and TAC levels increased with pregnancy progression or significantly raised during the 2nd and 3rd trimesters, and NLA of PON1, TOS, OSI, MDA, and HCY significantly increased before delivery in the ART and control groups. Compared with the control women, the ART women had substantially higher or relatively high lactonase activity and NLA of PON1 and TAC during pregnancy; higher triglyceride (TG), total cholesterol, low-density lipoprotein cholesterol, atherogenic index, apolipoprotein (apo) B, and apoB/apoA1 ratio in the 1st trimester; and higher fasting glucose, fasting insulin, homeostatic model assessment of insulin resistance, and TG levels before delivery. No significant differences were found in the frequencies of *PON1* C-108T and Q192R genotypes and alleles between the ART and control groups.

**Conclusions:**

Women with AMA undergoing ART had higher TAC, PON1 lactonase activity, and PON1 NLA than control women, suggesting increased compensatory antioxidant capacity in ART women, thus showing higher sensitivity to oxidative stress-related injury and diseases.

## Background

With the development of the modern economy and the improvement of female educational attainment, childbearing at advanced maternal age (AMA), defined as a pregnant woman with an estimated delivery date when she is 35 years old or older, has increased worldwide [1–3]. Childbearing at AMA leads to decreased fertility, an increased need for artificial reproductive technology (ART), and an increased risk for multiple pregnancy complications [2, 4]. Furthermore, developments in ART also contribute to an increased incidence of pregnancies in women outside the usual biological reproductive age [2] and result in a higher prevalence of multiple pregnancies, placenta previa, placental abruption or adherence, ante-partum or postpartum hemorrhage, preeclampsia (PE), preterm rupture of membrane, preterm birth, gestational diabetes mellitus (GDM), small for gestational age, and perinatal mortality [5, 6]. Additionally, the operation of ART itself and the precursor reason for seeking the help of ART are also risk factors for increasing the prevalence of complications during pregnancy [5, 7]. However, the underline pathophysiology remains unclear.

Oxidative stress is an imbalance between pro-oxidant molecules and antioxidant defenses, resulting from an increase in the levels of reactive oxygen species (ROS) and/or reactive nitrogen species (RNS) and/or a decrease or relative inadequacy in the antioxidant defense ability [8, 9]. Normal pregnancy is a series of complex endocrine-metabolic coordination and is well-known to increase the oxidative stress mainly produced by a normal systemic inflammatory response, which is of particular interest [8]. Oxidative stress has been recognized in various pathological disorders related to pregnancy, such as spontaneous abortion, preterm birth, GDM, and PE [8, 10–13].

Paraoxonase (PON) 1 is mainly synthesized in the liver and binds to high-density lipoprotein (HDL) granules in the blood circulation [11, 14, 15]. It is a calcium-dependent multifunctional enzyme that has arylesterase (AREase), paraoxonase (POase), lactonase, and Hcy-thiolactonase (HTase) activities [11, 15, 16]. PON1 protects against xenobiotic toxicity by hydrolyzing organophosphorus insecticides and several nerve agents. It has anti-oxidative, anti-inflammatory, anti-apoptosis, and anti-atherogenic properties by degrading oxidized lipids and homocysteine thiolactone, suppressing the oxidation of low-density lipoprotein (LDL), and diminishing the formation of macrophage foam cells [15–17]. Furthermore, several studies have proved that PON1 can degrade oxidized lipids through its lactonase activity [15, 18] and decomposes homocysteine thiolactone, which can increase the homocysteinylation of proteins and lead to protein inactivation and cell damage, through its HTase activity [16]. Therefore, the measurement of PON1 lactonase and HTase activities can better evaluate its anti-oxidative, anti-inflammatory, and anti-atherogenic characteristics compared to the tests of PON1 POase and AREase activities [11, 15, 16, 19].

Oxidative stress is involved in the pathophysiological processes of pregnancy [8, 10–13, 20]. PON1 is one of the necessary antioxidant enzymes in circulation and crucial in regulating the metabolism of oxidized lipids and homocysteine (HCY) [12, 14–16]. Our previous study found that increased oxidative stress and a compensatory elevation of PON1 level and lactonase activity are present in women with GDM and their neonates [11, 12]. Single-nucleoid polymorphism (SNP) of the *PON1* gene, such as C-108T and Q192R SNPs, has an evident impact on the levels and activities of PON1 [11, 12, 21]. However, to date, the lactonase activity and status of PON1 and the change of oxidative stress in women undergoing ART and its relationship with underlying pathophysiological mechanism remain unclear. In this study, we investigated the longitudinal changes of oxidative stress, HCY, the lactonase activity and status of PON1, and metabolic indexes. We analyzed the genotype distribution of *PON1* C-108T and Q192R SNPs in older women undergoing ART, which would possibly help to understand the reasons for the high prevalence of oxidative stress-related diseases in ART women.

## Methods

### Study participants

The present study was designed as a prospective nested case-control study. Study participants were recruited from the Department of Obstetrics and Gynecology of West China Second University Hospital between Sep.2017 and Apr.2020. The participants were ≥ 35 years at delivery, with singleton pregnancies, and delivered by cesarean sections. The study was approved by the Institutional Review Committee of West China Second Hospital of Sichuan University (No. 2017- 033 to Xinghui Liu and No. 2020-036 to Ping Fan). All participants provided written informed consent before the commencement of the study.

According to the mode of conception (ART vs. spontaneous), the participants were divided into ART and control groups and were excluded if they met one of the following criteria: (i) twin/multiple pregnancies; (ii) pre-pregnancy diabetes, chronic hypertension, GDM, hypertension in pregnancy, preeclampsia, intrahepatic cholestasis in pregnancy, endocrine disorders, cardiovascular diseases, autoimmune diseases, infections, and tumors; (iii) vaginal deliveries or emergency cesarean sections; (iv) long-term taking medication due to internal and external diseases during the study; (v) women that had premature delivery were also excluded from the control group.

All participants in this study had not taken any other medications except vitamins, minerals and trace elements for pregnant women (Elevit) and calcium (Caltrate) regularly during pregnancy.

Blood samples were obtained in the morning after overnight fasting at early pregnancy (gestational age 12–14 weeks), middle pregnancy (gestational age 24–26 weeks), late pregnancy (gestational age 32–34 weeks), and before cesarean section, respectively. The transportation and processing of samples were carried out at 4 °C. Serum and plasma aliquots were stored at -40 °C until analysis.

Clinical information, including age, pre-pregnancy body mass index (BMI, kg/m^2^), BMI before delivery, weight gain during pregnancy, systolic blood pressure (SBP), diastolic blood pressure (DBP), gestational age, and birth height and weight of infants of these mothers, were recorded.

### Analysis of PON1 activities, oxidative stress, and metabolic parameters

The lactonase activity and level of PON1 were analyzed using 5-thiobutyl butyrolactone (TBBL) and 7-O-diethylphosphoryl-3-cyano-4-methyl-7-hydroxycoumarin (DEPCyMC) as substrates, respectively, based on the dynamic methods as previously described [21]. The normalized lactonase activity (NLA) of PON1 was calculated using the following formula [19, 21]:

$$NLA\, = \,TBBL\,activity\,(U/mL)\, \times \,1000\,/\,DEPCyMC\,activity\,(mU/mL)$$

Serum malondialdehyde (MDA), total antioxidant capacity (TAC), total oxidant status (TOS), plasma fasting insulin (FIns) and glucose (FGlu), apolipoprotein (apo)A1, apoB, total cholesterol (TC), triglyceride (TG), LDL-cholesterol (LDL-C), HDL-cholesterol (HDL-C), atherosclerosis index (AI), oxidative stress index (OSI), the homeostatic model assessment of insulin resistance (HOMA-IR) were determined or assessed as previously described [9, 12, 22]. Serum HCY levels were measured by an enzymatic method (Shanghai Zhicheng Biological Technology Co., Ltd., Shanghai) using a Hitachi 7600-010 automatic analyzer.

All tests’ intra- and inter-assay variations were < 5% and 10%, respectively.

### Analysis of *PON1* C-108T and Q192R SNPs

The genomic DNA was isolated from peripheral blood leukocytes of the participants and *PON1* C-108T and Q192R SNPs were genotyped by polymerase chain reaction amplification and restriction fragment length polymorphism as previously described [12, 23] with some modifications for the measurement of Q192R SNP. Briefly, the polymerase chain reaction (PCR) primers of the Q192R polymorphism were designed using Primer-BLAST, and a 205-bp fragment was amplified using the forward primer: 5′-TATTGTTGCTGTGGGACCTGAG-3′, and reverse primer: 5′-AGAGTTCACATACTTGCCATCG-3′. The PCR products were digested with AlwI (New England Biolabs, Inc.). Digestion resulted in 64 and 141 bp fragments for the 192R allele and a non-digested 205 bp fragment for the 192Q allele. For genotyping quality control, we re-genotyped more than 30% of the DNA samples by a different operator, and all re-genotypes were consistent.

### Statistical analysis

Statistical Program for Social Sciences (SPSS) 26.0 was used to conduct all statistical analyses. The data were presented as mean ± standard deviation (SD) or relative frequencies (%), and differences between groups/subgroups were assessed using an independent sample t-test, one-way analysis of variance (ANOVA), or chi-squared (χ^2^) test. The differences in relevant indicators between the ART and control groups after adjusting for age and pre-pregnancy BMI were estimated using an analysis of covariance. The changes in the indicators with time were analyzed using one-way repeated measures ANOVA. Statistical significance was set at two-sided *P* < 0.05.

The NLA of PON1 was one of the main indexes in this study. A statistical power value was calculated based on sample size and the NLA of PON1 during the 1st, 2nd, and 3rd trimesters and before delivery by the Power and Sample Size Calculations (PS) Program Version 3.1.6.

## Results

### Clinical characteristics of the ART and control groups

The ART group consisted of women with infertility due to fallopian tubes factors (45.31%, 29 of 64), male infertility factors (12.50%, 8 of 64), undetermined factors (17.19%, 11 of 64), and other factors including pelvic adhesions, polycystic ovary syndrome, endometriosis, etc. (25.00%, 16 of 64).

As shown in Table 1, compared with the control group, the ART group had significantly lower gestation age, gravidity, parity, and neonatal weight (*P* < 0.05) but higher SBP (*P* = 0.008). There was no significant difference in age, pre-pregnancy BMI, BMI before delivery, weight gain during pregnancy, DBP, and neonatal length between the two groups (*P* > 0.05).

**Table 1:** Clinical characteristics in ART and control groups

	Controls (n = 129)	ART (n = 64)	*P*
Age (years)	37.16 ± 1.87	37.22 ± 2.34	0.857
Pre-pregnancy BMI (kg/m^2^)	21.59 ± 2.45	21.32 ± 2.57	0.472
BMI before delivery (kg/m^2^)	27.10 ± 2.30	26.39 ± 2.65	0.057
Weight gain during pregnancy (kg)	13.87 ± 3.73	12.96 ± 3.73	0.115
Gestation age (days)	275.22 ± 3.73	271.44 ± 8.53	0.001
Gravidity	3.22 ± 1.45	2.59 ± 1.64	0.007
Parity	1.74 ± 0.49	1.14 ± 0.35	< 0.001
SBP (mmHg)	113.78 ± 10.79	118.14 ± 10.48	0.008
DBP (mmHg)	71.90 ± 7.15	73.66 ± 7.58	0.117
Neonatal birth length (cm)	50.01 ± 2.24	49.81 ± 2.00	0.556
Neonatal birth weight (g)	3395.70 ± 342.87	3275.47 ± 422.81	0.036

Values are presented as the mean ± SD

*Abbreviations*: *ART* artificial reproductive technology, *BMI* body mass index, *DBP* diastolic blood pressure, *SBP* systolic blood pressure

### Metabolic parameters between the ART and control groups during different pregnant periods

Metabolic parameters between the ART and control women during the 1st, 2nd, and 3rd trimesters and before pregnancy delivery are shown in Table 2.

**Table 2:** Metabolic parameters in ART and control groups during different pregnant periods

	1st trimester	2nd trimester	3rd trimester	delivery	P
**Control (n = 129)**					
FGlu (mmol/L)	4.40 ± 0.25	4.34 ± 0.19	4.31 ± 0.21^a^	4.34 ± 0.32	0.038
FIns (pmol/L)	7.24 ± 5.07	10.55 ± 10.47^a^	11.52 ± 9.37^a^	8.67 ± 5.16^c^	< 0.001
HOMA-IR	1.42 ± 1.01	2.04 ± 2.08^a^	2.22 ± 1.90^a^	1.69 ± 0.99^c^	< 0.001
TG (mmol/L)	1.57 ± 0.47	2.65 ± 0.88^a^	3.39 ± 1.19^a, b^	3.66 ± 1.47^a, b, c^	< 0.001
TC (mmol/L)	4.44 ± 0.56	5.64 ± 0.81^a^	5.96 ± 0.86^a, b^	5.85 ± 1.10^a, b^	< 0.001
HDL-C (mmol/L)	1.77 ± 0.33	2.06 ± 0.42^a^	1.99 ± 0.39^a, b^	1.82 ± 0.39^b, c^	< 0.001
LDL-C (mmol/L)	2.22 ± 0.58	3.06 ± 0.83^a^	3.33 ± 0.94^a, b^	3.18 ± 1.00^a^	< 0.001
AI	1.59 ± 0.52	1.81 ± 0.52^a^	2.07 ± 0.56^a, b^	2.28 ± 0.59^a, b, c^	< 0.001
Apo A1 (g/L)	2.13 ± 0.34	2.53 ± 0.52^a^	2.45 ± 0.41^a^	2.35 ± 0.51^a. b^	< 0.001
ApoB (g/L)	0.74 ± 0.16	0.99 ± 0.20^a^	1.11 ± 0.21^a, b^	1.12 ± 0.27^a, b^	< 0.001
ApoB/apoA1	0.36 ± 0.11	0.41 ± 0.12^a^	0.47 ± 0.13^a, b^	0.49 ± 0.14^a, b, c^	< 0.001
**ART (n = 64)**					
FGlu (mmol/L)	4.39 ± 0.21	4.33 ± 0.18	4.34 ± 0.20	4.56 ± 0.34*^, a, b, c^	< 0.001
FIns (pmol/L)	7.13 ± 3.49	8.94 ± 4.65^a^	10.88 ± 5.32^a, b^	11.85 ± 10.89*^, a, b^	< 0.001
HOMA-IR	1.39 ± 0.68	1.72 ± 0.88^a^	2.09 ± 1.02^a, b^	2.40 ± 2.20*^, a, b^	< 0.001
TG (mmol/L)	1.72 ± 0.57*	2.77 ± 0.94^a^	3.64 ± 1.34^a, b^	4.34 ± 2.05*^, a, b, c^	< 0.001
TC (mmol/L)	4.66 ± 0.63*	5.85 ± 0.86^a^	6.12 ± 0.94^a^	6.16 ± 1.15^a^	< 0.001
HDL-C (mmol/L)	1.73 ± 0.35	2.06 ± 0.43^a^	1.96 ± 0.39^a, b^	1.83 ± 0.37^b, c^	< 0.001
LDL-C (mmol/L)	2.45 ± 0.68*	3.30 ± 0.80^a^	3.50 ± 0.94^a^	3.30 ± 0.96^a^	< 0.001
AI	1.79 ± 0.63*	1.93 ± 0.55	2.20 ± 0.60^a, b^	2.42 ± 0.66^a, b, c^	< 0.001
Apo A1 (g/L)	2.10 ± 0.37	2.55 ± 0.49^a^	2.46 ± 0.49^a^	2.30 ± 0.44^a, b^	< 0.001
ApoB (g/L)	0.79 ± 0.17*	1.03 ± 0.21^a^	1.14 ± 0.26^a, b^	1.17 ± 0.30^a, b^	< 0.001
ApoB/apoA1	0.39 ± 0.12*	0.42 ± 0.12^a^	0.49 ± 0.16^a, b^	0.53 ± 0.16^a, b^	< 0.001

Values are presented as the mean ± SD

*Abbreviations*: *AI* atherosclerosis index, *ApoA1* apolipoprotein A1, *ApoB* apolipoprotein B, *ART* artificial reproductive technology, *FGlu* fasting glucose, *FIns* fasting insulin, *HDL-C* high-density lipoprotein cholesterol, *HOMA-IR* homeostasis model assessment of insulin resistance, *LDL-C* low-density lipoprotein cholesterol, *TC* total cholesterol, *TG* triglycerides

All comparisons of parameters were corrected for differences in age and pre-pregnancy BMI between the ART and control groups

*P < 0.05, compared with the control corresponding subgroup; ^a^*P* < 0.05, compared with 1st trimester subgroup; ^b^*P* < 0.05, compared with 2nd trimester subgroup; ^c^*P* < 0.05, compared with 3rd trimester subgroup

In the ART and control groups, TG, AI, and apoB/apoA1 ratio gradually increased with pregnancy progression (*P* < 0.001); TC, HDL-C, LDL-C, apoA1, apoB, FIns, and HOMA-IR reached a peak during 2nd and 3rd trimester, and then became stable or slightly declined before delivery (*P* < 0.001). In addition, FGlu was lower in 3rd trimester than in 1st trimester (*P* = 0.038) in the control group and was highest before delivery in the ART group (*P* < 0.001).

After correcting for differences in age and pre-pregnancy BMI between the ART and control groups, TG, TC, LDL-C, AI, apoB, and apoB/apoA1 ratio in the 1st trimester and the FGlu, FIns, HOMA-IR, and TG levels before delivery were significantly higher in the ART women than in the control women (*P* < 0.05).

### Oxidative stress indexes between the ART and control groups in different pregnant periods

Oxidative stress parameters between the ART and control groups during the 1st, 2nd, and 3rd trimesters and pregnancy delivery are shown in Table 3.

**Table 3:** Oxidative stress parameters and PON1 activities in ART and control groups during different pregnant periods

	1st trimester	2nd trimester	3rd trimester	delivery	*P*
**Control (n = 129)**					
TOS (µmol H_2_O_2_ Equiv./L)	16.26 ± 10.64	15.40 ± 8.82	16.09 ± 6.70	22.76 ± 13.32^a, b, c^	< 0.001
TAC (mmol Trolox Equiv./L)	0.96 ± 0.18	1.05 ± 0.15^a^	1.07 ± 0.16^a^	1.02 ± 0.24	< 0.001
OSI	15.03 ± 6.76	13.72 ± 4.49	15.19 ± 5.68^b^	18.03 ± 8.35^a, b, c^	< 0.001
MDA (nmol/mL)	4.84 ± 1.67	5.08 ± 1.52	5.09 ± 1.26	6.02 ± 2.25^a, b, c^	< 0.001
HCY (µmol/L)	6.72 ± 1.25	6.60 ± 1.22	6.92 ± 1.26^b^	8.88 ± 2.26^a, b, c^	< 0.001
PON1 lactonase activity (U/mL)	16.20 ± 2.44	15.52 ± 2.64^a^	15.21 ± 2.18^a^	14.81 ± 2.36^a, b, c^	< 0.001
PON1 level (mU/mL)	42.38 ± 6.57	40.45 ± 6.20^a^	39.26 ± 5.78^a, b^	36.53 ± 6.02^a, b, c^	< 0.001
NLA	384.50 ± 40.44	383.98 ± 49.21	389.40 ± 40.07^a^	408.07 ± 43.64^a, b, c^	< 0.001
**ART (n = 64)**					
TOS (µmol H2O2 Equiv./L)	14.73 ± 5.04	15.34 ± 5.02	16.62 ± 3.77	24.33 ± 12.39^a, b, c^	< 0.001
TAC (mmol Trolox Equiv./L)	1.04 ± 0.18*	1.12 ± 0.15*^, a^	1.12 ± 0.17*^, a^	1.08 ± 0.25	0.033
OSI	14.21 ± 5.91	13.73 ± 4.40	15.40 ± 5.19	18.32 ± 6.83^a, b, c^	< 0.001
MDA (nmol/mL)	4.65 ± 1.33	4.88 ± 0.86	5.30 ± 1.49^a^	5.66 ± 1.64^a, b^	< 0.001
HCY (µmol/L)	7.12 ± 2.05	6.73 ± 1.17	7.25 ± 1.34	8.81 ± 2.47^a, b, c^	0.001
PON1 lactonase activity (U/mL)	16.92 ± 3.24	16.48 ± 2.90*	16.20 ± 3.02*^, a^	15.84 ± 3.23*^, a^	< 0.001
PON1 level (mU/mL)	42.58 ± 8.84	41.26 ± 7.86	40.16 ± 7.90^a, b^	37.18 ± 7.44^a, b, c^	< 0.001
NLA	401.34 ± 48.32*	403.09 ± 49.29*	406.92 ± 48.80*	428.39 ± 47.89*^, a, b, c^	< 0.001

Values are presented as the mean ± SD

*Abbreviations*: *ART* artificial reproductive technology, *HCY* homocysteine, *MDA* malondialdehyde, *NLA* normalized lactonase activity, *OSI* oxidative stress index, *PON1* paraoxonase 1, *TAC* total antioxidant capacity, *TOS* total oxidant status

All comparisons of parameters were corrected for differences in age and pre-pregnancy BMI between the ART and control subgroups

*P < 0.05, compared with the control corresponding subgroup; ^a^*P* < 0.05, compared with 1st trimester subgroup; ^b^*P* < 0.05, compared with 2nd trimester subgroup; ^c^*P* < 0.05, compared with 3rd trimester subgroup

**Fig. 1:**
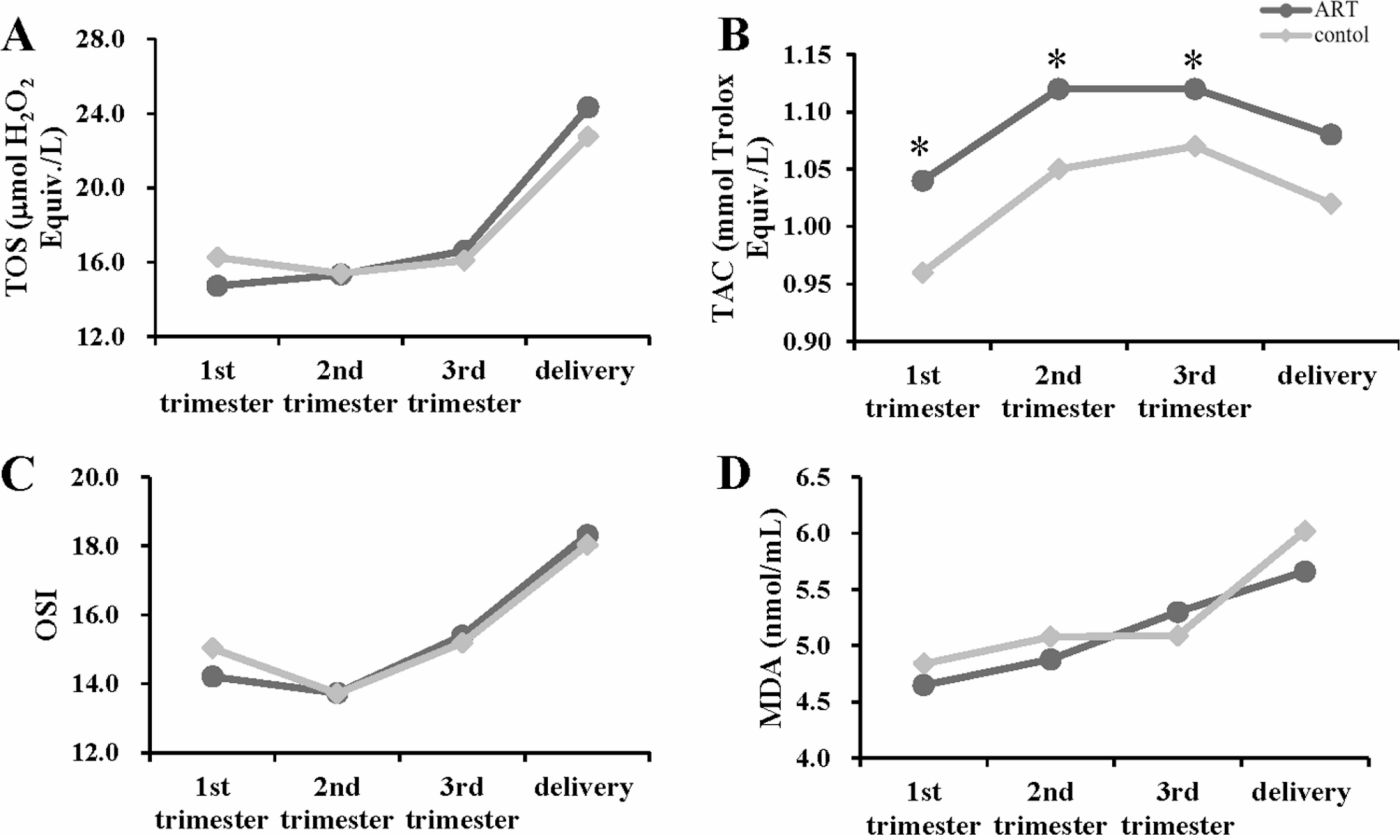
Longitudinal changing tendency of oxidative stress parameters. Longitudinal changes in oxidative stress parameters during gestation in the ART and control women. (**A**) total oxidant status (TOS); (**B**) total antioxidant capacity (TAC); (**C**) oxidative stress index (OSI); (**D**) malondialdehyde (MDA). *P < 0.05, compared with the control corresponding subgroup

**Fig. 2:**
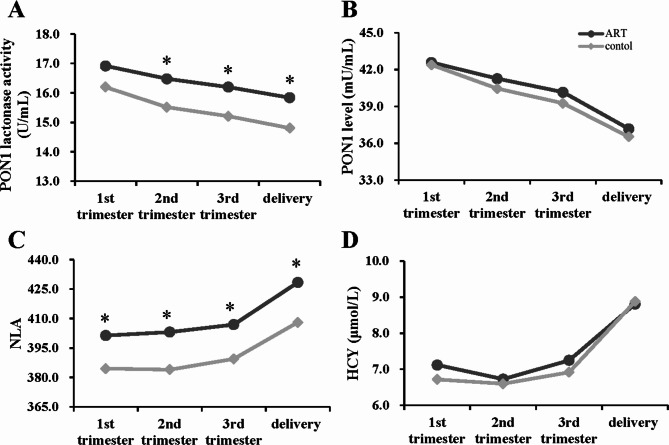
Longitudinal changing tendency of PON1 activities and HCY levels. Longitudinal changes in the lactonase activity, level and normalized lactonase activity (NLA) of paraoxonase 1 (PON1), as well as homocysteine (HCY) level during gestation in the ART and control women. (**A**) PON1 lactonase activity; (**B**) PON1 levels; (**C**) PON1 NLA; (**D**) HCY. *P < 0.05, compared with the control corresponding subgroup

In the ART and control groups, serum TOS, OSI, MDA, and HCY levels were significantly higher before delivery than in the 1st, 2nd, and 3rd trimesters (*P* < 0.001), while TAC levels were significantly higher in the 2nd and 3rd trimesters than in the 1st trimester (*P* < 0.05; Table 3; Fig. 1, and Fig. 2D).

Serum TAC levels in the 1st, 2nd, and 3rd trimesters were substantially higher in the ART women than in control women after correcting for differences in age and pre-pregnancy BMI (*P* < 0.05; Table 3, and Fig. 1B).

### Lactonase activity and status of PON1 during different pregnant periods and PON1 *C-108T* and *Q192R* SNPs in the ART and control groups

As shown in Table 3; Fig. 2A-C, the lactonase activity and level of PON1 gradually decreased with pregnancy progression. They fell to the lowest levels before delivery, while the NLA of PON1 reached its peak before delivery in both ART and control groups (*P* < 0.001).

The lactonase activity of PON1 in the 2nd and 3rd trimesters and before delivery; and the NLA of PON1 in the 1st, 2nd, and 3rd trimesters and before delivery were significantly higher in the ART group than in the control group after correcting for differences in age and pre-pregnancy BMI (*P* < 0.05; Table 3; Fig. 2A-C). The statistical power values of 0.707, 0.813, 0.741, and 0.820 were achieved in the study for the NLA of PON1 during the 1st, 2nd, and 3rd trimesters and before delivery, respectively (within-group standard deviations = 43.78, 43.69, 43.78, and 46.02, respectively; differences in the case and control means = 16.84, 19.11, 17.52, 20.32, respectively; significance level = 0.05).

The genotype distributions of the *PON1* C-108T and Q192R SNPs were in Hardy‒Weinberg equilibrium in both ART and control groups (all *P* > 0.05). No significant differences were observed in the frequencies of the *PON1* C-108T and Q192R genotypes and alleles between the ART and control groups (*P* > 0.05, Table 4).

**Table 4:** Frequencies of PON1 C-108T and Q192R genotypes and alleles in ART and control groups

		Controls (n = 124)	ART (n = 53)	*x* ^*2*^	*P*
**C-108T**					
Genotype	CC	39 (31.5%)	17 (32.1%)		
	CT	56 (45.2%)	23 (43.4%)		
	TT	29 (23.4%)	13 (24.5%)	0.051	0.975
Allele	C	134 (54.0%)	57 (53.8%)		
	T	114 (46.0%)	49 (46.2%)	0.002	0.964
**Q192R**					
Genotype	QQ	13 (10.5%)	8 (15.1%)		
	QR	56 (45.2%)	22 (41.5%)		
	RR	55 (44.4%)	23 (43.4%)	0.785	0.675
Allele	Q	82 (33.1%)	38 (35.8%)		
	R	166 (66.9%)	68 (64.2%)	0.257	0.612

Values are presented as number (%) of women undergoing IVF or control women

## Discussion

To our knowledge, this is the first study to investigate the longitudinal changes of oxidative stress and PON1 lactonase activity and status during gestation in women undergoing ART. We found that the lactonase activity and level of PON1 gradually decreased with pregnancy progression. However, TAC levels significantly increased during the 2nd and 3rd trimesters. NLA of PON1 and oxidative stress parameters, including TOS, OSI, MDA, and HCY, significantly increased in the ART and control groups before delivery. Moreover, we found that the lactonase activity of PON1 in the 2nd and 3rd trimesters and before delivery, the NLA of PON1 in the 1st, 2nd, and 3rd trimesters and before delivery, and TAC in the 1st, 2nd, and 3rd trimesters were significantly higher in the ART women than in the control women. Besides, we also showed that the women undergoing ART had an adverse lipid metabolic profile in the 1st trimester and a higher FGlu, FIns, HOMA-IR, and TG levels before delivery compared with the control women. We consider that an unfavorable glycolipid metabolism and a compensatory increase in PON1 activities and TAC during pregnancy in the women undergoing ART may be the partial reasons that hindered a natural conception and increased the risk of metabolic and oxidative stress-related diseases including pregnancy complications in these women.

Pregnancy is a stress state related to an atherogenic lipid profile in women [11, 24]. Pregnancy produces transient insulin resistance manifested as elevated postprandial glucose and fasting lipid levels, increased inflammatory response, and enlarged circulating blood volume [25]. Maternal metabolic changes during early pregnancy are mostly increasing lipid synthesis and body fat accumulation from hyperphagia arising in response to a hormonal stimulus [26, 27]. On the other hand, again due to hormones, there can be an initial weight loss due to hyperemesis. As pregnancy progresses, this accumulation stops or even declines during the 3rd trimester as a consequence of both enhanced lipolysis and decreased fatty acid intake in adipose tissue, which in turn causes the increases of fatty acids into the liver, the enhancement of very low-density lipoprotein synthesis and secretion, and the elevation of lipoprotein levels in circulation [27]. Meanwhile, placental hormones and other mediators facilitate peripheral insulin resistance [26]. It was previously reported that a 40–50% reduction in insulin sensitivity and a compensation of 200–250% increase in insulin secretion by pancreatic β cells to maintain maternal euglycemia [28]. Consistent with previous reports [29, 30], in this study, we showed that TG, TC, and LDL-C levels gradually increased with pregnancy progression and peaked during the 3rd trimester or before delivery in the ART and control groups. We further demonstrated that the longitudinal changes of HDL-C, AI, apoA1, apoB, FIns, apoB/apoA1 ratio, and HOMA-IR during gestation also represented similar trends. However, unlike the tendency of physiological changes in the control women, the FGlu, FIns, and HOMA-IR during gestation reached the highest levels before delivery in the ART group. The TG, TC, LDL-C, AI, apoB, and apoB/apoA1 ratio in the 1st trimester and the FGlu, FIns, HOMA-IR, and TG before delivery were significantly higher in the ART women than in the control women. Our findings suggest that ART women have an adverse lipid metabolic profile and a fragile balance in glucose metabolism, corroborating previous reports that women undergoing ART have a higher risk of GDM [6, 31].

Another characteristic of pregnancy stress is increased oxidative stress [11, 24, 32]. Implantation and placentation in early pregnancy resemble “an open wound in the uterus”, and the following inflammatory environment is essential for repairing the uterine epithelium and removing cellular debris resulting from blastocysts and trophoblasts [33]. The systematic inflammation results in increased oxidative stress in the 1st trimester and maintained a redox balance in the 2nd and 3rd trimesters for rapid fetal growth and developed again until delivery, which needs a strong inflammatory response to promote uterine contractions and delivery of baby and placenta [33, 34]. Pereira et al. [34] reported increasing oxidative stress levels in the placenta caused by high mitochondrial activity and increased ROS production. Measurement of TOS and TAC can assess the total amount of oxidant and antioxidant molecules present in serum, respectively [9, 11]. OSI, the ratio of TOS to TAC, can estimate the redox state of the body [9]. MDA is an end-product of lipid peroxidation and the hallmark of ROS-induced injury [35]. A study has reported an increased TAC and TOS during the 3rd trimester and before delivery in normal pregnancy [32]. This study showed that TOS, OSI, and MDA levels significantly increased before delivery. However, TAC increased and peaked at 2nd or 3rd trimester and decreased before delivery in the ART and control groups.

Oxidative stress is involved in the pathophysiological process of female infertility. Diseases causing infertility, such as PCOS, obesity, advanced maternal age, and reproductive system inflammation, are associated with oxidative stress [34]. In addition, operations and medications in ART could lead to oxidation-reduction imbalance. A study reported lower superoxide dismutase and higher MDA and sulfhydryl groups after ovarian stimulation, indicating increased oxidative stress after ovarian stimulation [36]. Besides, applying gonadotropins in ART may generate large amounts of ROS and lead to oxidation-reduction imbalance, directly affecting maternal serum’s oxidative stress indicators, such as antioxidant capacity, susceptibility to oxidation in vitro, and antioxidants in vivo [37]. Our findings showed that the ART women had significantly higher TAC during pregnancy than the control women, suggesting a compensatory increase in antioxidant capacity exists in these women.

PON1 plays antioxidant and anti-atherogenic roles by hydrolyzing lipid peroxides and homocysteine thiolactone (HTL) depending on its lactonase and HTase activities [15, 16, 19, 38]. HCY, along with HTL, is an essential precursor of oxidative stress, activating autoimmune, enhancing thrombosis response, and interacting with LDL to form foam cells [16, 38]. Studies found a significant decrease in serum POase activity of PON1 during the 3rd trimester and before delivery in normal pregnancy [30, 32]. In this study, we found that the lactonase activity and level of PON1 gradually decreased with pregnancy progression. However, the NLA of PON1 and HCY levels significantly increased before delivery in the ART and control groups. Furthermore, we found that the ART women had significantly higher lactonase activity and NLA of PON1 during pregnancy compared with the control women, suggesting that a compensatory increase in PON1 activities in these women.

PON1 activities and status are influenced by various factors, such as genetic polymorphisms of PON1, oxidative stress, lipoproteins, and apolipoproteins [11, 21, 39, 40]. Genetic variants of *PON1* play vital roles in regulating the expression and/or activities of PON1 and may explain more than 60% of the individual differences in enzyme protein levels and activities [12, 21, 41]. Among PON1 SNPs known, the SNP C-108T, a binding site for the transcription factor Sp1 in the promoter region, has the greatest impact on the gene expression of PON1 and can account for 22.8% of the observed variability [21, 42, 43]. The SNP Q192R, a common variation in the exon region, mainly affects the enzyme activities of PON1 in a substrate-dependent manner [11, 15, 44]. The Q isoform hydrolyzes soman, sarin, diazoxon, and lipid peroxides more efficiently [15, 44], whereas the R isoform hydrolyzes paraoxon (POase activity) more rapidly in vitro [44, 45]. Oxidative stress has a reversible effect on the levels and activities of PON1. Previous studies found that serum TAC and MDA levels correlated positively with the lactonase activity and levels of PON1, respectively, suggesting that the elevated TAC, PON1 lactonase activity, and PON1 levels might compensate for increased oxidative stress in women with GDM and their neonates [11, 12]. However, hyperglycemia and excessive oxidant molecules may cause glycosylation and oxidative damage to PON1, reducing enzyme activity [46–48]. PON1 activities are also affected by apolipoproteins. Both apoA1 and apoE combined with PON1 in HDL could help stimulate the PON1 lactonase activity and strengthen the enzyme stability [39, 41]. In this study, the lactonase activity and NLA of PON1 were significantly higher at different stages of pregnancy in the ART group than in the control group. Consequently, the genotypic frequencies of the *PON1* C-108T and Q192R polymorphisms were not significant differences between the ART and control groups, suggesting that the compensatory increase in PON1 activities might be mainly related to an unfavorable state of glycolipid metabolism and oxidative stress.

Some limitations need to be acknowledged. First, the participants in this study are limited to women with AMA; however, age might affect oxidative stress and metabolic indicators and was an independent risk factor for many pregnancy complications. A comparison between young and AMA women would help figure out the compact of age. Second, this was a single-center study, and we could not include more participants. Limited by the relatively small sample size, we failed to perform subgroup analyses, for example, reasons for undergoing ART and different technologies such as in vitro fertilization and embryo transfer, intracytoplasmic sperm injection, and preimplantation genetic diagnosis. Third, we did not genotype *PON1* polymorphisms in some participants (ART = 11; control = 5) due to a missing DNA sample, which might have affected the power of these parameters.

## Conclusions

For the first time, the present study systematically detected oxidative stress indicators and the lactonase activity and status of PON1 at different time points in gestation in ART women. We found that women with AMA undergoing ART had higher TAC, PON1 lactonase activity, and PON1 NLA than the control women. This suggests that a compensatory increase in antioxidant capacity exists in ART women and thus might be more sensitive to oxidative stress-related injury and diseases. Our findings also provide evidence that ART women may develop an unfavorable glycolipid metabolism. Whether these changes potentially cause metabolic disorders later in the life of ART women and their offspring remains to be determined. Further research could focus on early pregnancy interventions that could help improve prognosis and prevent diseases. Moreover, the health status of these women and their offspring require other clinical follow-ups.

## Data Availability

The datasets used and/or analyzed during the current study are available from the corresponding author upon reasonable request.

## Abbreviations

AI: atherosclerosis index
AMA: advanced maternal age
Apo: apolipoprotein
AREase: arylesterase
ART: artificial reproductive technology
BMI: body mass index
DBP: diastolic blood pressure
DEPCyMC: 7-O-diethylphosphoryl-3-cyano-4-methyl-7-hydroxycoumarin
FGlu: fasting glucose
FIns: fasting insulin
GDM: gestational diabetes mellitus
HCY: homocysteine
HDL: high-density lipoprotein
HOMA-IR: homeostatic model assessment of insulin resistance
HTase: Hcy-thiolactonase
LDL: low-density lipoprotein
MDA: malondialdehyde
NLA: normalized lactonase activity
OSI: oxidative stress index
PE: preeclampsia
POase: paraoxonase
PON1: paraoxonase 1
RNS: reactive nitrogen species
ROS: reactive oxygen species
SBP: systolic blood pressure
SNP: single-nucleoid polymorphism
TAC: total antioxidant capacity
TBBL: 5-thiobutyl butyrolactone
TOS: total oxidant status
